# Frequency of Piroplasms *Babesia microti* and *Cytauxzoon felis* in Stray Cats from Northern Italy

**DOI:** 10.1155/2014/943754

**Published:** 2014-05-04

**Authors:** Eva Spada, Daniela Proverbio, Paola Galluzzo, Roberta Perego, Giada Bagnagatti De Giorgi, Nora Roggero, Santo Caracappa

**Affiliations:** ^1^Reparto di Medicina Emotrasfusionale Veterinaria (REV), Dipartimento di Scienze Veterinarie per la Salute, La Produzione Animale e la Sicurezza Alimentare (VESPA), Università degli Studi di Milano, Via G. Celoria, 10-20133 Milano, Italy; ^2^Centro di Referenza Nazionale per Anaplasma, Babesia, Rickettsia e Theileria (C.R.A.Ba.R.T.), Istituto Zooprofilattico Sperimentale della Sicilia, Via G. Marinuzzi, 3-90129 Palermo, Italy

## Abstract

Emerging diseases caused by piroplasms pose a health risk for man and other animals, and domestic cats have been proposed as potential reservoirs for some piroplasm infections. The aim of this study was to identify the frequency of the piroplasms *Babesia microti* and *Cytauxzoon felis* in stray cats from northern Italy and to identify possible risk factors associated with these infections. Blood samples from 260 stray cats enrolled in a trap-neuter-release (TNR) program in northern Italy were examined with conventional PCR for the presence of *Babesia microti* and *Cytauxzoon felis* DNA. No sample (0.0%) tested positive for *C. felis*, whilst *B. microti* DNA was detected in two samples (0.8%). Both infected cats were in good clinical condition and recovered well from the neutering surgery. One of these two cats had a triple coinfection with *Babesia microti*, *Candidatus* Mycoplasma haemominutum, and *Anaplasma phagocytophilum*. Evidence presented in this study indicates that the blood borne protozoans *Babesia microti* and *Cytauxzoon felis* are not widely distributed in stray cat populations in Milan, northern Italy, and that the significance of cats as a reservoir host for *B. microti* in this area is limited.

## 1. Introduction

Members of the order Piroplasmida (such as the genera* Babesia* and* Cytauxzoon*) are apicomplexan protozoa. They live in the blood cells of vertebrates and are transmitted between hosts by ticks and could also be transmitted by iatrogenic (e.g., blood transfusion) and congenital transmission. Emerging diseases caused by some of these piroplasms pose health risks for man and other animals [1].

*Babesia microti*, a babesial parasite of rodents, has been identified as the cause of more than 300 cases of human babesiosis since 1969, causing disease ranging from mild to severe in nature [2]. Pennisi et al. (2007) [3] identified six blood samples from owned pet cats from southern Italy (Sicily) that tested positive for* Babesia microti* and proposed that cats might be a reservoir for this parasite.

Dogs and cats live in close proximity to people and have often been proposed as effective sentinel animals to assess the risk of human infection with tick-borne diseases [4]. This is particularly true for feral and stray cats that roam freely, are often exposed to potential tick vectors and to wildlife reservoirs, have a poor nutritional and clinical status, and receive no prophylactic treatment against ectoparasites. In Italy ticks, that can also affect people, have been found on cats (e.g.,* Ixodes ricinus* and* Rhipicephalus sanguineus*) [5] but the veterinary significance of this parasitism is largely unknown [6]. The lack of knowledge about the distribution and transmission of zoonotic diseases in stray cats (which may be potential reservoirs for infection) is delaying the implementation of effective prophylactic and control measures to limit the spread of these infections in the pet and human populations.

*Cytauxzoon felis *is a protozoan hemoparasite of wild and domestic cats that is transmitted between feral and domestic animal populations by ticks [1]. The transmission of* C. felis* infection from one domestic cat to another by* Amblyomma americanum* ticks confirms that these ticks are the primary vectors for this infection [7]. However, it is hypothesized that other ticks such as* Ixodes ricinus* and* Dermacentor* sp. may be involved in the transmission of* Cytauxzoon *sp. infection [8, 9]. Because of the continued emergence of* C. felis* worldwide [9, 10] and the geographical expansion of the tick vector, it is likely that* C. felis* will increasingly be found in areas not currently recognized as enzootic for this protozoan parasite. In addition, with the detection of a high prevalence of erythroparasitemia predominately in apparently healthy cats and the evidence of persistence of infection after recovery, several authors [9, 11, 12] propose that domestic cats may serve as a reservoir host for* Cytauxzoon* sp. infection and thus significantly increase the risk of exposure for other cats.

Although not documented, it is theoretically possible that cytauxzoonosis could be transmitted between domestic cats during blood transfusion, since some cats have persistent blood parasitemia without clinical illness and cats that survive clinical cytauxzoonosis may remain persistently infected [12]. The administration of blood products to cats has become routine in veterinary practice. Candidate donors should be screened for infectious diseases to minimize the risk of disease transmission through blood transfusions. However, the decision on which infectious agents to screen for in canine and feline transfusion medicine depends on the prevalence of the relevant agents in the regions where the donor lives or visits. Data on the prevalence of vector-borne infectious diseases such as cytauxzoonosis in stray cats are also useful for differential diagnosis in sick owned pets that share the same environment and infected ticks as the feral population.

The aim of the present study was to identify the presence and frequency of the piroplasms* Babesia microti* and* Cytauxzoon felis* in stray cats in northern Italy, by means of conventional PCR, and to identify possible risk factors associated with these infections.

## 2. Material and Methods

### 2.1. Study Area

The study was carried out in Milan (coordinates: 45°28′N, 09°11′E), an industrial, commercial, and financial city (181.76 km^2^) and capital of the Lombardy region of northern Italy. The city is located in the north-western section of the Po Valley, approximately halfway between the river Po, to the south, and the first reliefs of the Alps, with the Great Lakes to the north, the Ticino river to the west, and the Adda river to the east. The municipal territory is entirely flat, with the highest point being 122 m above sea level. According to the Köppen climate classification, Milan has a humid, subtropical climate, in which moderately hot summers and cold humid winters prevail.

The wild mammals present in Milan city are small, including the brown rat (*Rattus norvegicus*), house mouse (*Mus musculus*), European hedgehog (*Erinaceus europaeus*), and Kuhl's pipistrelle (*Pipistrellus kuhlii*) [13].

In Milan city there is a large population of stray cats, with more than 500 feline colonies [14] which are controlled through a no-kill trap-neuter-release (TNR) program intended to limit the reproduction of free-roaming cats.

### 2.2. Sample Population and Data Collection

During a 2-year period (from January 2008 to January 2010), blood samples were collected from 260 feral cats from urban colonies in Milan, northern Italy, during a TNR program approved by the local authority of the city council as previously described [15].

Briefly, cats trapped by volunteers and delivered to the University of Milan were anesthetized with a combination of tiletamine and zolazepam (12 mg/kg Zoletil 100, Virbac, Italy) plus tramadol (1 mg/kg, Altadol, Formevet, Italy), given intramuscularly, based on estimated body weight, while cats were confined in the trap. General anesthesia was maintained with isoflurane (Isoflo, Esteve, Italy) given by mask.

### 2.3. Signalment and Health Status of the Cats

The information collected on each cat included age (estimated based on dentition; animals ≤ 6 months of age were considered juvenile, whereas all others were considered adult), gender (male or female), breed, and data obtained from physical examination (healthy or unhealthy). Unhealthy cats were defined as cats with the presence of one or more of the following clinical abnormalities: lymph node enlargement, pale mucous membranes, stomatitis, or signs of ocular and respiratory infections. Body condition score (BCS) was also recorded [16]. Cats were examined for the presence of ectoparasites (ticks or fleas).

### 2.4. Hematological Analysis

Blood samples were collected aseptically from the jugular vein during anesthesia and placed in tubes with EDTA anticoagulant. Within 24 h of sample collection, EDTA anticoagulated blood was evaluated for a complete blood count (CBC) using an ADVIA 120 System (Siemens Healthcare Diagnostics, Milan, Italy). EDTA blood surplus was stored at −20°C until PCR analysis.

### 2.5. PCR Analysis for* Babesia microti* and* Cytauxzoon felis*

#### 2.5.1. Genomic DNA Extraction

DNA was extracted directly from blood samples using a commercial DNA extraction kit (PureLink Genomic DNA Mini Kit, Invitrogen, Carlsbad, CA, USA). Extracted DNA was stored at −20°C.

#### 2.5.2. Primers and PCR Assay

Two pairs of primers, ITS2F (5′-TGAACGTATTAGACACACCACCT-3′) and ITS2R (5′-TCCTCCCGCTTCACTCGCCG 3′) and BAB1 (5′-CTTAGTATAAGCTTTTATACAGC-3′) and BAB4 (5′- ATAGGTCAGAAACTTGAATGATACA-3′), were used to amplify ITS and 18S rRNA regions of* Cytauxzoon felis* and* Babesia microti*, respectively. The amplicon expected size was 231 bp for* Babesia microti* and 431 bp for* Cytauxzoon felis* [17, 18].

The reaction mixture included 2 *μ*L of template DNA, 0.25 mM dNTPs, 0.4 mM of each primer, 1X reaction buffer, and 2.5 U* Taq* DNA polymerase (GoTaq DNA Polymerase, Promega, Madison, WI, USA). The volume of this mixture was adjusted to 25 *μ*L with sterile water. PCR conditions have been previously reported [17, 18]. PCR products were resolved using 1.5% agarose gel and fragment size was estimated using a DNA molecular weight marker (50 bp DNA Ladder; Promega, Madison, WI, USA).

#### 2.5.3. Sequence Confirmation

*B. microti* positive products were purified using GFX PCR DNA and gel band purification kit (GE Healthcare Life Sciences, Buckinghamshire, UK) in accordance with the manufacturer's protocol. Purified samples were used for sequencing reactions carried out using a Big Dye Terminator v.3.1 Cycle Sequencing Kit (Life Technologies, Carlsbad, CA, USA). The sequencing reaction product was purified using an Illustra Autoseq G-50 Dye Terminator Removal Kit (GE Healthcare Life Sciences, Buckinghamshire, UK) and 2 *μ*L of sample was analyzed using an ABI3130 Genetic Analyzer (Life Technologies, Carlsbad, CA, USA). Finally, the nucleotide sequences were analyzed by BLAST software and compared with sequences present in GenBank.

## 3. Results

All the cats were domestic shorthair, 45.4% (118/260) were juvenile, and 54.6% (142/260) were adult. Ninety cats (34.6%) were male, and 170 (65.4%) were female. BCS was recorded in 243 cats, 18 cats (7.4%) were underweight (BCS 1–3/9), and 225 cats (92.6%) had normal weight (BCS 4–6/9). The health status was evaluated in all cats, 72 cats (27.7%) were healthy, and 188 (72.3%) were unhealthy and showed the following clinical abnormalities: lymph node enlargement (133 cats, 70.7%), pale mucous membranes (14 cats, 7.4%), stomatitis (101 cats, 53.7%), signs of respiratory tract infection (22 cats, 11.7%), and signs of ocular infection (40 cats, 21.3%). The CBC abnormalities were recorded in 150 cats and the results are as follows: anemia (Ht < 24%) in 69 cats (46.0%), leukopenia (WBC < 10,570/*μ*L) in 14 cats (9.3%), leukocytosis (WBC > 14,390/*μ*L) in five cats (3.3%), and thrombocytopenia (PLT < 200,670/*μ*L) in 10 cats (6.7%). Ectoparasites were recorded for 149 cats with fleas present in 25 cats (16.8%) and ticks in four cats (2.7%).

Two of 260 blood samples (0.8%, 95% CI 0.093–2.778) tested positive for* B. microti* DNA and no blood samples (0.0%) tested positive for* C. felis*. The gene accession bank confirmed these two samples as* Babesia microti* (GenBank accession number EU882727).

Of the 2 cats infected by* B. microti*, one was an adult, entire female domestic shorthair with a good BCS (4/9) weighing 3.2 kg. This cat was found to be infested with fleas but no ticks were present. She was in good clinical condition despite a mild generalized lymph node enlargement and stomatitis. This cat also tested PCR positive for* Candidatus* M. haemominutum and* A. phagocytophilum* in a previous survey [19]. Recovery from surgery and hospitalization was uneventful in this animal.

The second* B. microti* PCR positive cat was an adult, entire male domestic shorthair. This cat also had a normal weight and BCS (4.1 kg and 5/9, resp.) and no ectoparasites were found. This cat was in good general clinical condition, with only mild enlargement of the popliteal lymph nodes. Its recovery from surgery and hospitalization was unremarkable.

## 4. Discussion

The present study represents the first report of the prevalence of* Babesia microti* in stray cats from northern Italy and only the second survey of* B. microti* and* C. felis* infection in cats in Italy. In addition, this is the first report of a triple coinfection with* B. microti*,* Candidatus* M. haemominutum, and* A. phagocytophilum* in a cat.

The first confirmed autochthonous case of* B. microti* infection in a human being was reported in Europe in 2007 [20] and several studies have demonstrated the presence of* B. microti* isolates in* Ixodes ricinus* ticks removed from dogs and cats in Europe [21, 22]. Moreover, a recent study has indicated the presence of zoonotic* B. microti* in rodents in Croatia [23]. Therefore, it is likely that* B. microti* infection in people occurs more often in Europe than was previously recognized.

The only data on the prevalence of* B. microti* in domestic cats comes from the study of Pennisi et al. [3] in which the blood of 6 out of 23 cats was found to be PCR positive for this pathogen. The results of our* B. microti* survey indicate that the prevalence of infection in stray cats in northern Italy is very low, less than 1% of the sampled population, and therefore stray cats seem to be a limited reservoir for* B. microti* infection and not a significant source of infection for the ticks in this area. The free-roaming, hunting behavior of stray cats increases their exposure to the potentially infected sylvatic tick populations present on rodents which act as the natural reservoir for* B. microti*. Domestic mice (*Mus musculus*) are documented reservoirs for* B. microti* in Europe [24] and are present in Milan city [13]. It is likely that this was the source of infection for the two infected cats in this study.

Both* B. microti* positive cats had no ticks at the time of diagnosis. Both had mild clinical abnormalities on examination (lymphadenopathy) but were in good clinical condition and recovered well from surgery and hospitalization. These findings support a theory that cats are incidental hosts for* Babesia microti* and survive the infection.

One cat showed a triple coinfection with* B. microti*,* Candidatus* M. haemominutum, and* A. phagocytophilum*. This is not the first report of domestic cats with multiple vector-borne infections. Recently Maia et al. (2013) [25] described the first detection of* Cytauxzoon felis* and* Candidatus* M. haemominutum coinfection in a Brazilian domestic cat in South America.

*C. felis* infection was not found in our stray cat population. Data from our survey are in agreement with the low prevalence (0.8% in 116 domestic cats) reported in a study in France [26] and 0.3% positivity in 961 free-roaming cats in the USA [11] but are in contrast to data on the first endemic focus of* Cytauxzoon* sp. found recently in northeastern Italy [9] in which 23% of 118 domestic and stray cats tested PCR positive and to a study in an endemic area of the USA which showed a positivity of 30.3% in 89 domestic cats [12].

The differences between the results of the present study and those of the highly endemic area for* C. felis* in Trieste in northeastern Italy [9] could be explained by the fact that the cats surveyed in Trieste lived in an urban area near wooded land which represents a preferred habitat for the tick vectors. The presence of large wild animals such as roe deer, foxes, and Eurasian lynx near Trieste increases the survival rate of ticks and helps to transport them to new areas, thus potentially increasing the spread of* C. felis* to new hosts. These observations support the finding that these hemoprotozoa occur in hyperendemic foci or “hot spots” within the same country as previously demonstrated in the USA [11].

The results of this study suggest that, unlike other blood borne infections such as* Mycoplasma* spp.,* Ehrlichia *spp.,* Anaplasma phagocytophilum*, and* Rickettsia* spp., for which there is a moderate-high risk of infection in stray cats from Milan city [19, 27], there is no need to screen blood donor cats for* C. felis* infection in this area.

A limitation of our study is that we did not investigate whether the strains of* B. microti* isolated from the two cats were human-infecting strains and genetic characterizations of previous samples of* B. microti* have indicated that both zoonotic and presumed nonzoonotic strains can cocirculate in the same species of rodents [23]. Another limitation is that the blood samples were collected between 2008 and 2010, so our results may no longer be valid, if the situation has now changed (four years later).

## 5. Conclusion

This study demonstrates that the hemoprotozoa* Babesia microti* and* Cytauxzoon felis* are not widely distributed in stray cat populations of Milan, in northern Italy. Domestic cats are likely to provide a very limited reservoir for* B. microti* in this area, but more studies are needed to confirm the reservoir competence of domestic cats for* B. microti*.
